# First evidence of dengue infection in domestic dogs living in different ecological settings in Thailand

**DOI:** 10.1371/journal.pone.0180013

**Published:** 2017-08-30

**Authors:** Suporn Thongyuan, Pattamaporn Kittayapong

**Affiliations:** 1 Center of Excellence for Vectors and Vector-Borne Diseases, Faculty of Science, Mahidol University at Salaya, Nakhon Pathom, Thailand; 2 Department of Biology, Faculty of Science, Mahidol University, Bangkok, Thailand; University of California Davis, UNITED STATES

## Abstract

**Background:**

Dengue is a vector-borne disease transmitted by *Aedes* mosquitoes. It is considered an important public health problem in many countries worldwide. However, only a few studies have been conducted on primates and domestic animals that could potentially be a reservoir of dengue viruses. Since domestic dogs share both habitats and vectors with humans, this study aimed to investigate whether domestic dogs living in different ecological settings in dengue endemic areas in Thailand could be naturally infected with dengue viruses.

**Methodology/Principal findings:**

Serum samples were collected from domestic dogs in three different ecological settings of Thailand: urban dengue endemic areas of Nakhon Sawan Province; rubber plantation areas of Rayong Province; and Koh Chang, an island tourist spot of Trat Province. These samples were screened for dengue viral genome by using semi-nested RT-PCR. Positive samples were then inoculated in mosquito and dog cell lines for virus isolation. Supernatant collected from cell culture was tested for the presence of dengue viral genome by semi-nested RT-PCR, then double-strand DNA products were double-pass custom-sequenced. Partial nucleotide sequences were aligned with the sequences already recorded in GenBank, and a phylogenetic tree was constructed. In the urban setting, 632 domestic dog serum samples were screened for dengue virus genome by RT-PCR, and six samples (0.95%) tested positive for dengue virus. Four out of six dengue viruses from positive samples were successfully isolated. Dengue virus serotype 2 and serotype 3 were found to have circulated in domestic dog populations. One of 153 samples (0.65%) collected from the rubber plantation area showed a PCR-positive result, and dengue serotype 3 was successfully isolated. Partial gene phylogeny revealed that the isolated dengue viruses were closely related to those strains circulating in human populations. None of the 71 samples collected from the island tourist spot showed a positive result.

**Conclusions/Significance:**

We concluded that domestic dogs can be infected with dengue virus strains circulating in dengue endemic areas. The role of domestic dogs in dengue transmission needs to be further investigated, i.e., whether they are potential reservoirs or incidental hosts of dengue viruses.

## Introduction

Dengue virus (DENV), transmitted by *Aedes* mosquitoes, is the major arbovirus threat to public health worldwide [1–3]. A licensed dengue vaccine has been used for dengue prevention in several countries, including Thailand, but evidence indicated that it conferred only partial protection against DENV infection. Prospective strategies for dengue prevention and control should focus on development of highly effective dengue vaccine together with integrated vector management [4–9]. Four serotypes of DENV circulate in both endemic (humans serve as both reservoirs and amplifying hosts and peri-domestic *Aedes* mosquitoes serve as vectors) and sylvatic (non-human primates serve as reservoir hosts and forest-dwelling *Aedes* mosquitoes serve as vectors) cycles [10–11].

Although many studies have focused on detection of DENV in human hosts and mosquitoes, only a few studies have been done on primates and domestic animals which could potentially be reservoirs of this virus. A study in Malaysia reported a high level of antibody against dengue in wild monkeys [12], and studies on animal models as carriers of dengue indicated that monkeys could be infected by DENV with no clinical signs present [13,14]. There was evidence of DENV RNA detected in the brains of bats caught from a dengue endemic area in Hainan Island in China [15] and Mexico [16,17], and detection of antibodies against DENV in bats collected from Costa Rica, Ecuador [18], the Yucatan peninsula of Mexico [19]; as well as detection of antibodies against DENV serotype 2 and a DENV genome in neotropical forest mammals in French Guiana that was closely related to those strains circulating in human populations [20,21]. However, there has been no investigation into whether these mammals are incidental hosts or potential reservoirs of DENV [22].

Domestic dogs play a vital role in human societies. They share human environment, and some live in houses together with their owners. They are not only pets but also viewed as family members. This relationship facilitates increased opportunities to pass or exchange pathogens between humans, animals, and vectors. Although domestic animals are identified as reservoirs of many zoonotic diseases, since they share both habitats and vectors with humans [23,24], it has been difficult to diagnose these viral infections in animals. However, there have been several studies on domestic dogs in relation to flavivirus surveillance, such as West Nile virus (WNV), which stated that dogs could be useful sentinels for monitoring areas with evidence of WNV [25–27], and also Japanese encephalitis virus (JEV) [28,29], since they typically show no clinical signs even when infected and viremia is developed [25,29,30]. Studies on blood feeding patterns of *Aedes aegypti* and *Aedes albopictus* collected from dengue endemic areas, such as Thailand, Singapore, United States of America and Puerto Rico, illustrated that these dengue-transmitting mosquitoes could also feed on domestic dogs [31–35]. This evidence supports our hypothesis that domestic dogs can be involved in dengue transmission cycle. Our study therefore aimed to determine whether DENV could naturally infect domestic dogs living in a dengue endemic area in three different ecological settings in Thailand (urban city, rubber plantation, and island tourist spot).

## Materials and methods

### Study sites

**Fig 1:**
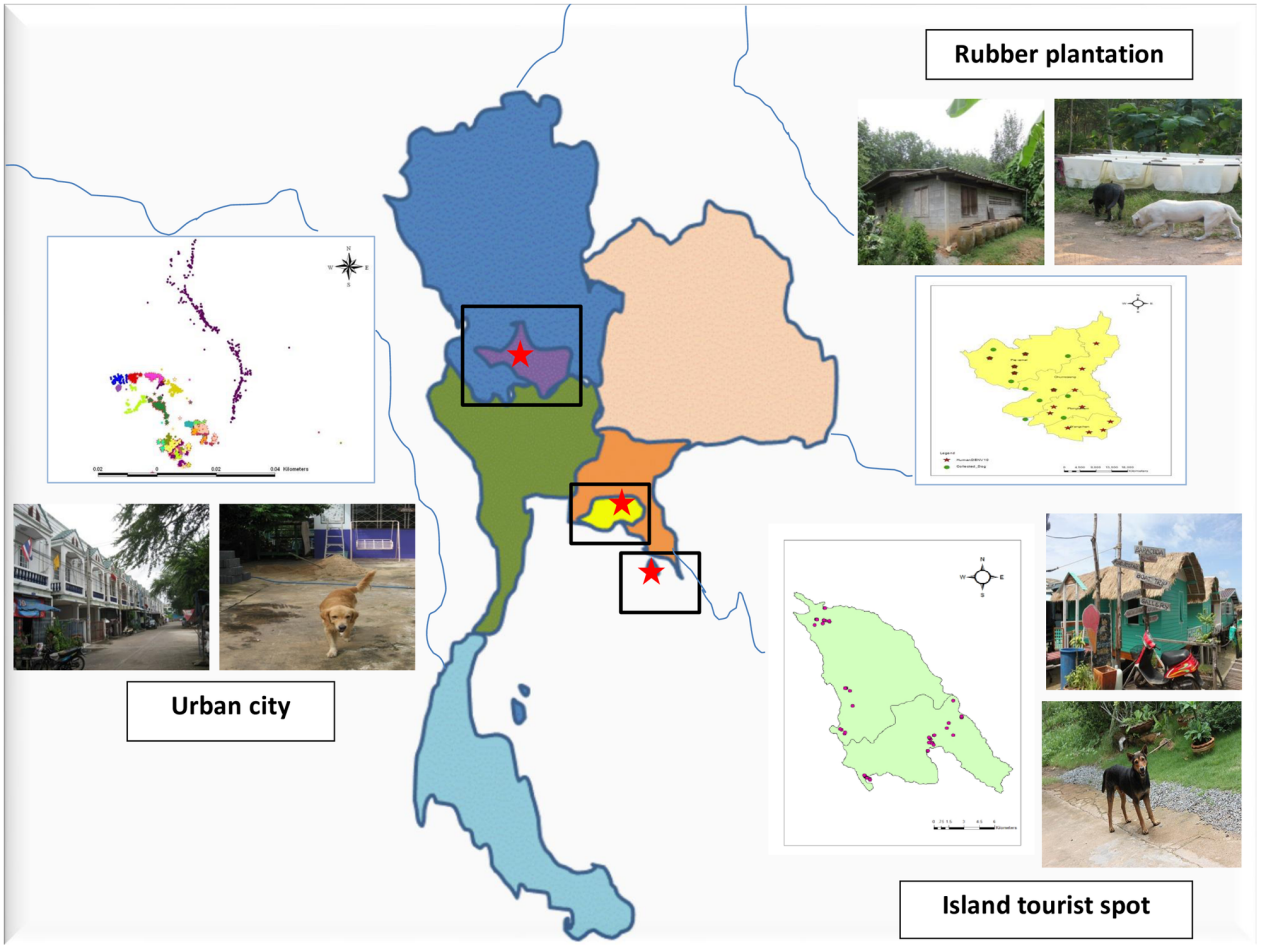
Map of Thailand highlighting locations of three sampling sites in the three different ecological settings: Urban city, rubber plantation area and an island tourist spot.

We conducted our study in three different ecological settings within a dengue endemic area in Thailand: urban city, rubber plantation area, and island tourist spot (Table 1 and Fig 1). The Muang District of Nakhon Sawan Province, located in the upper central part of Thailand (15°42ˊ18˝ N, 100°8ˊ15˝ E), was identified as an urban city area. This area has been affected by dengue and has kept Nakhon Sawan Province on the top ten ranking of dengue epidemic areas in Thailand for many years. Houses located in 15 communities of this area were visited twice, from September to December 2008 and during a twelve-month followed-up period from September to December 2009. These communities have a variety of houses in densely populated areas, including commercial and residential areas, which also are the habitats of domestic dogs. Wang Chan District, Rayong Province, located in the eastern part of Thailand (12°56ˊ5˝ N, 101°31ˊ13˝ E), is a rubber plantation area. Dengue has been endemic in this area, and there was also an outbreak of chikungunya among residents during 2009. Houses in this area are located in or close to rubber plantations. We visited these houses in September 2009 and then at a six-month follow-up period in March 2010. The island tourist spot of Koh Chang, Trat Province, is located in the eastern part of Thailand (12°6ˊ13˝ N, 102°21ˊ7˝ E) and is composed of residential areas for both tourists and local people, forested areas, and agricultural land. Dengue has been reported throughout the year but with low morbidity rate. Thirty houses of permanent residence in three communities on Koh Chang were visited during January and June of 2012.

**Table 1:** Distribution of collected dog serum samples and RT-PCR positive results.

Settings	Dates of collection	No. of houses visited	No. of collected samples	RT-PCR positive samples (%)
**Urban city**	Sep-Dec 2008	1,138	632	6 (0.95)
	Sep-Dec 2009	414	425	0
		Totals	1,057	6 (0.57)
**Rubber plantation area**	Sep 2009	59	153	1 (0.65)
	Mar 2010	16	21	0
		Totals	174	1 (0.57)
**Island tourist spot**	Jan 2012	30	38	0
	Jun 2012	20	33	0
		Totals	71	0

### Ethical considerations

All animal experiments in this study was carried out in strict accordance with the recommendations in the Guide for the Care and Use of the National Laboratory Animal Center of Thailand. Owners of animals signed a written consent form before blood collection. In addition, well-trained veterinarians, with gentle restraint to minimize suffering, performed blood collection.

The animal experimental protocol was reviewed and approved by the Institutional Animal Care and Use Committee (IACUC) of the Faculty of Science, Mahidol University, Thailand (Approval Protocol Numbers: MUSC56-018-280 and MUSC56-023-285/2).

### Collection of serum samples

We collected blood samples from all available domestic dogs living in the households of each ecological setting for further analysis. Specific information relating to domestic dogs (such as breed, age, sex, health status, housing, and environment) was recorded for sample identification. Blood samples were collected without anti-coagulant by veterinarians. One to three milliliters of blood was collected by veni-puncture, then centrifuged, and the serum was frozen at -80°C until used.

### Laboratory diagnosis of serum samples

#### RNA extraction

The genomic viral RNA was extracted from 100 μL of domestic dog serum sample and/or virus infected cell culture supernatants by using Trizol (GIBCO-BRL, Rockville, MD, USA), according to the manufacturer’s instructions.

#### Genome detection assay

The RT-PCR was performed according to the protocol developed by Lanciotti et al. [36], a semi-nested RT-PCR targeting the Capsid and pre-Membrane (C/prM) region. The positive control was RNA extracted from DENV-positive human serum donated by the Center of Excellence for Vectors and Vector-Borne Diseases, Faculty of Science, Mahidol University, while nuclease-free water was used as a negative control. In all steps of PCR, avoiding contamination was strictly managed. The process had been done respectively with negative control, samples and positive control. A portion of the reaction products of first and second rounds of PCR were observed directly in 2% agarose gel stained with ethidium bromide. If the result of the first round of PCR was shown as expected, then the second round could be started. The use of filter tips, disposal pipettes including UV irradiation of PCR working areas and all equipment was strictly performed. The size of the resulting DNA band characterized for each DENV type was 482, 119, 290 and 392 base pairs for DENV serotypes 1–4 respectively.

#### Viral isolation in mammalian and mosquito cell cultures

A confluent monolayer of dog (MDCK) and mosquito (C6/36) cell lines were grown in 24-well culture plates (NUNC, Roskilde, Denmark) in Dulbecco's Modified Eagle's Medium (DMEM) supplemented with 10% fetal bovine serum (FBS) and penicillin (100 unit/mL), streptomycin (100 μg/mL), and nystatin (20 μg/mL). The cell culture medium and reagents were procured from Sigma (St. Louis, MO, USA). DENV PCR-positive sera were processed for virus isolation according to the standard protocol. The sera were diluted (1:10) in DMEM supplemented with penicillin, streptomycin, and nystatin. One hundred microliters of the filtered sera were inoculated in the confluent monolayer of MDCK and C6/36 cells and incubated for adsorption at 37°C for 1 hour for MDCK and at 28°C for C6/36. After adsorption, the cells were washed with PBS (pH7.5) and fed with fresh DMEM supplemented with 2% FBS. Inoculated cells were kept in a CO_2_ incubator supplying 95% air and 5% CO_2_. Appropriate cell controls, which were un-inoculated cell cultures, were maintained in a similar manner. Virus infected cells were blindly passed to a new flask every seven days for 21 days. The cells were observed daily with an inverted microscope for any cytopathic effects (CPE) for 21 days or 3 passages. Two hundred microliters of supernatant fluids were collected at days 3, 7, 14, and 21 after inoculation. Cell debris was removed by centrifugation, then the supernatant was filtered through 0.2 μm membrane and kept at -80°C until use. The presence of DENV genome was tested by RT-PCR as explained above.

#### Real-time PCR

Quantitative real-time PCR was performed by using CFX96^™^ Real-Time System (Bio-Rad) and Phire Tissue Direct PCR Master Mix Reagents (Thermo Scientific^®^), according to the manufacture’s instruction. The amount of virus particles contained in the supernatant collected from virus isolation had been determined by the in-house real-time PCR which had the limit of quantitation (LoQ) at 10^4^ copies of RNA equivalent viral genome per milliliters and the limit of detection (LoD) at 10^3^ copies/mL.

#### Phylogenetic analysis

The RT-PCR products of DENV-positive samples were purified by the Qiagen purification kit (Chatsworth-USA), and then they were double-pass custom-sequenced with a Big dye terminator cycle sequencing ready reaction kit (Applied Biosystems, USA) on an ABI310 sequencer following the manufacturer’s protocol. Sequences were edited using the Bioedit program. Then sequences of the isolates were submitted to GenBank. The BLAST program was used for the database search. The partial gene sequences were assembled and aligned using CLUSTALW version 1.83 [37] and were analyzed using phylogenetic software (MEGA 6.0) [38]. The phylogenetic tree was constructed by the maximum likelihood method with 1,000 bootstrap replications.

## Results

### Detection of DENV in serum samples from domestic dogs

**Fig 2:**
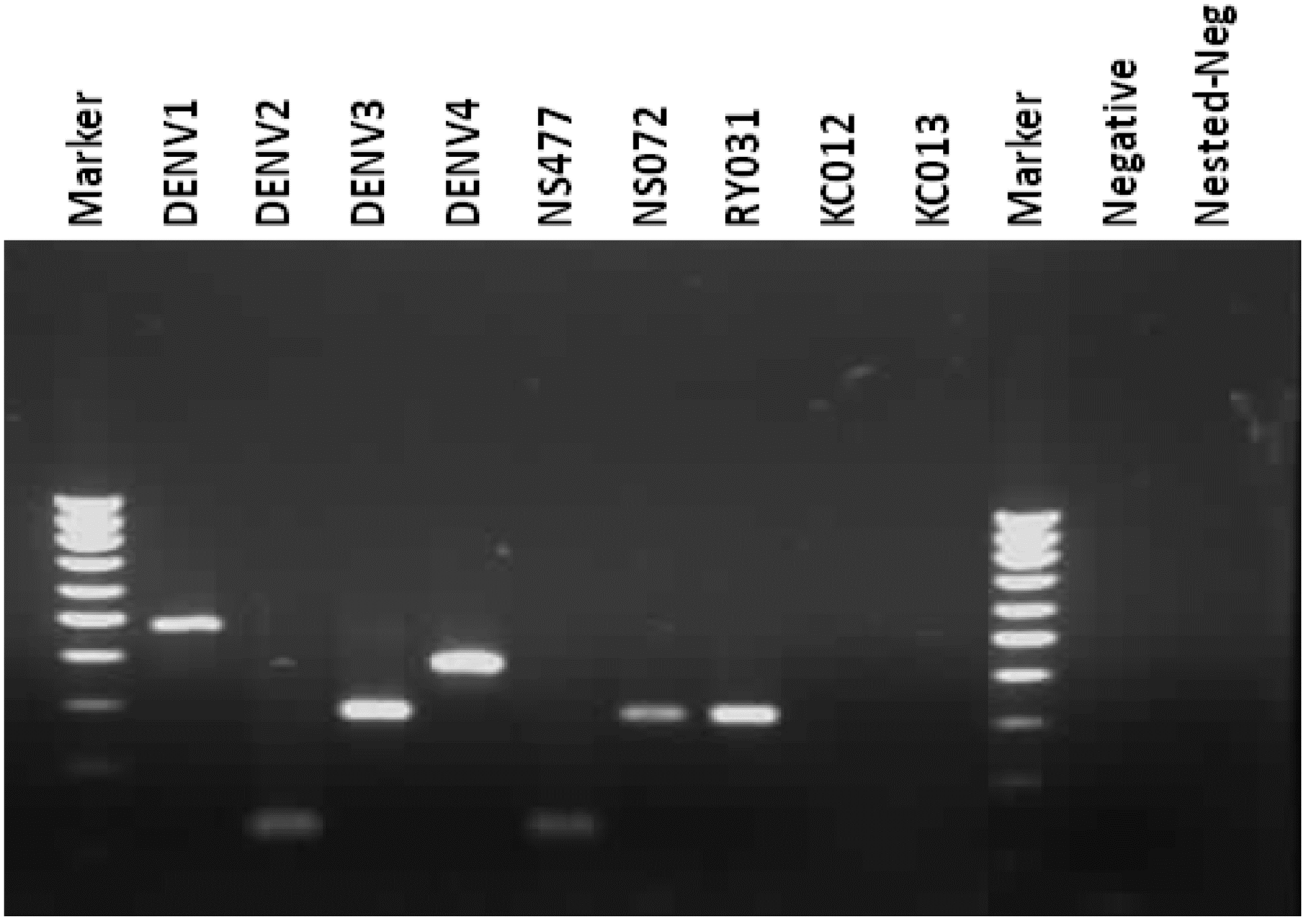
Nested RT-PCR of DENV genome detection in domestic dog serum samples. NS477 showed positive results for DENV serotype 2 while NS072 and RY031 showed positive results for DENV serotype 3.

Serum samples from domestic dogs living in three different ecological settings within a dengue endemic area in Thailand, i.e., urban city, rubber plantation area, and island tourist spot (Fig 1), were screened for DENVs. The distribution of collected dog serum samples screened for DENV are shown in Table 1. In total, 1,227 houses were visited and 1,302 blood samples of domestic dogs were collected. In the urban dengue endemic area, 6 out of 632 (0.95%) samples collected from September to December 2008 tested positive for DENV. Two samples tested positive for DENV serotype 2, while four samples were positive for DENV serotype 3. Fig 2 showed nested RT-PCR of dengue genome detection in domestic dog serum samples and positive results for DENV serotype 2 and serotype 3.

**Fig 3:**
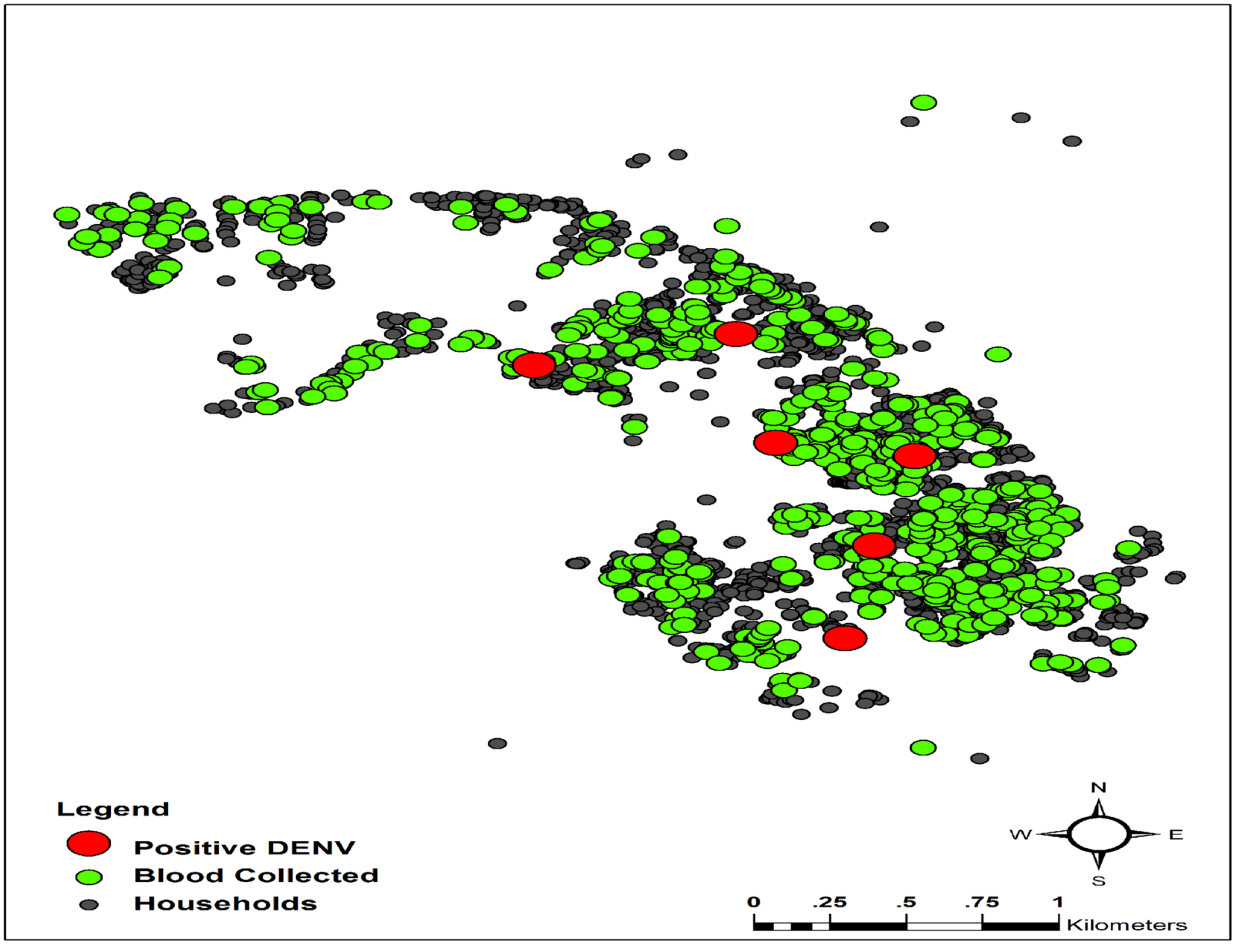
Urban setting area, highlighting locations of houses where dog serum samples were collected and locations of DENV-positive dogs.

The characteristics of DENV-positive dogs are shown in Table 2. All DENV-positive poodle breed dogs lived inside houses together with their owners. They usually walked and played in the houses and surroundings of their owners during the day-time, and did not go outside their household territories. Contrastingly, Thai breed dogs always lived outdoors, with no cage, and were free ranging. But they did not stray far from their houses, due to the dense situation of the urban area. The locations of houses in the urban city setting where dog serum samples were collected and found positive for DENV are shown in Fig 3.

**Table 2:** Characteristic of DENV-positive dogs.

Sample ID	Setting/Location	Breed	Sex	Age (month)	Habitat
**NS062**	Urban city/C1	Poodle	Male	72	Indoor
**NS072**	Urban city/C1	Thai	Female	72	Outdoor
**NS173**	Urban city/C3	Thai	Female	72	Outdoor
**NS255**	Urban city/C5	Poodle	Female	36	Indoor
**NS477**	Urban city/C15	Poodle	Male	48	Indoor
**NS596**	Urban city/C23	Poodle	Male	36	Indoor
**RY031**	Rubber plantation	Thai	Female	36	Outdoor

We visited 59 houses located in the rubber plantation area, and 153 domestic dogs were screened for DENV during September 2009. Only one sample tested positive for DENV, which was identified as DENV serotype 3. Domestic dogs living in this area were identified as free ranging. Normally, these domestic dogs spent time in and around the houses of their owners, while their playgrounds extended to the rubber plantation areas which became part of their habitats.

Domestic dogs living on the island tourist spot, Koh Chang, were also screened for DENV. Thirty houses of permanent residences were visited in January 2012, and 38 dog blood samples were collected. None of them tested positive for DENV. The dogs in this setting had no cage, and they lived both indoors and outdoors in proximity to the premises of their owners, which provided them more space when compared with those in the urban city setting, but less space when compared to those of the rubber plantation area.

### Virus isolation from DENV-positive serum samples

All seven DENV-positive serum samples of domestic dogs were inoculated into C6/36 and MDCK cell lines for viral isolation. In the C6/36 cell culture, four positive samples (NS062, NS072 NS477 and RY031) successfully infected and were grown in the cell lines. At day 21 after inoculation, DENV serotype 3 was identified in the supernatant collected from NS062, NS072 and RY031; and DENV serotype 2 was identified in the supernatant collected from NS477 (Table 3).

**Table 3:** Laboratory diagnostics of PCR-positive blood samples of domestic dogs.

		Cell culture		Sequencing	
**Sample ID**	RT-PCR	C6/36	MDCK	Serotype	Accession number
**NS062**	DEN3	DEN3	Neg	DEN3	KY820699
**NS072**	DEN3	DEN3	Neg	DEN3	KY820702
**NS173**	DEN2	Neg	Neg	NA	NA
**NS255**	DEN3	Neg	Neg	NA	NA
**NS477**	DEN2	DEN2	Neg	DEN2	KY82068
**NS596**	DEN3	Neg	DEN3	DEN3	KY820701
**RY031**	DEN3	DEN3	DEN3	DEN3	KY820700

Neg means Negative, NA means Not analyzed

In the MDCK cell line, only two DENV-positive serum samples (NS596 and RY031) were successfully infected and were grown. DENV was detected after amplification with specific primers by using RT-PCR at day 21 after inoculation. Supernatant collected from both blood samples was identified as DENV serotype 3 (Table 3).

In mosquito and dog cell lines, DENV genome was not found in supernatant collected on days 3, 7 and 14 after inoculation. Only RY031 successfully infected and was grown in both cell lines.

We performed in-house real-time PCR for dengue serotype assay targeting C/prM region. However, all supernatants showed negative result.

### Phylogenetic analysis

**Fig 4:**
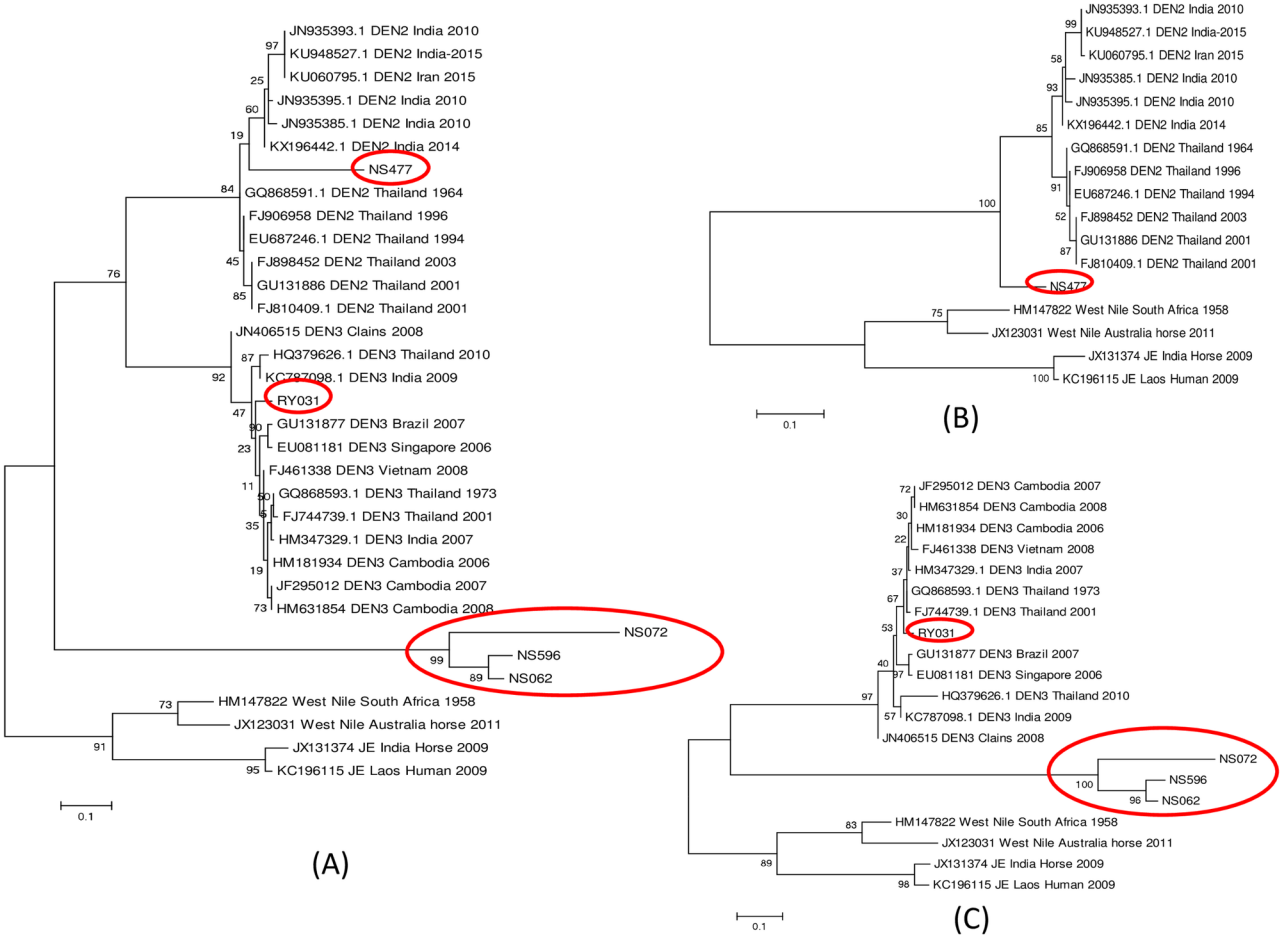
Phylogenetic analysis of DENV-positive dog serum isolates. The phylogenetic tree was derived from the partial nucleotide sequences of C/prM gene using maximum likelihood method with 1,000 bootstrap value. (A) DENV serotype 2 and 3, (B) DENV serotype 2, (C) DENV serotype 3. Domestic dog sequences are indicated in red circles.

Separate phylogenetic trees for the partial C/prM gene were constructed for DENV serotype 2 and DENV serotype 3 using the MEGA 6. Maximum likelihood phylogenetic trees are presented in Fig 4. One sequence of DENV serotype 2 (NS477) and four sequences of DENV serotype 3 (NS062, NS072, NS596 and RY031) which were isolated from domestic dogs were compared with 12 sequences of DENV serotype 2, twelve sequences of DENV serotype 3 and four sequences of WNV and JEV isolated from human populations obtained from GenBank (Fig 4A). These sequences were deposited in GenBank, the accession numbers are presented in Table 3. In total, 17 sequences were aligned for DENV serotype 2. The phylogenetic analysis showed that NS477 was closely related but separated from DENV serotype 2 which was isolated from human populations by 100% bootstrap value. However, the size of the sequence aligned is so small (<119 bp), that this relationship cannot be specified with any confidence (Fig 4B). For DENV serotype 3, twenty sequences were aligned; the phylogenetic analysis clearly showed that RY031 was closely related to DENV serotype 3 which were isolated from human populations, supported by 97% bootstrap value. However, we also found that the three urban domestic dog sequences (NS062, NS072, and NS596) were aligned in separate groups, supported by bootstrap value of 100% (Fig 4C).

## Discussion

Currently, diagnosis of DENV is based on serology, viral isolation, and RNA detection according to WHO guidelines [39], which states that the laboratory criteria for confirmation of dengue infection are: 1) isolation of the DENV; or 2) demonstration of a four-fold rise antibody; or 3) demonstration of DENV antigen; or 4) detection of DENV genomic sequences. RT-PCR was the most suitable test for this study, as it was able to detect DENV up to the 10^th^ day after the onset of the symptoms in humans [40,41]. Our study confirmed DENV infection in domestic dogs by detecting the DENV genome using RT-PCR together with an isolation of DENV by inoculation in the mosquito and dog cell lines. DENV serotype 2 and serotype 3 were identified circulating in domestic dog populations living in dengue endemic area. The dogs screened in this study were healthy, consistent with the findings in non-human primates [16,18].

Virus isolation by inoculating DENV into mosquito cell lines, particularly C6/36, has been routinely used due to its high sensitivity for DENV infection, likewise, in this study, 4 out of 6 samples were successfully isolated by C6/36 cell lines. Mammalian cell lines, such as monkey, baby hamster and dog kidney cell lines, also have been evaluated for susceptibility to DENV infection [39, 41–47]. In our study, DENV collected from two samples, NS596 and RY031, were successfully isolated by using dog cell lines (MDCK). These results support the idea that DENV can enter dog cells and that dog might serve as hosts for DENV. The susceptibility of domestic dogs to DENV should be further investigated. Viral load in clinical samples could play a critical role in amplifying virus in cell cultures or mosquitoes [46]. One sample, NS596 showed positive result only for MDCK, not C6/36. This might be the result of low level of virus replicated in C6/36 cell line or the variation of virus strain or technical error due to handling with low viral load in the serum sample. Additionally, we used a combination of virus isolation and RT-PCR to confirm DENV genome in filtered supernatants of inoculated cell lines rather than to observe CPE or IFA as RT-PCR is more precise and sensitive [46–51], especially when detection of the low viral load present in the serum sample [52]. Although, Indirect immunofluorescence assay (IFA) is routinely used in the detection of DENV antigen in infected cell lines, however, this technique is time consuming and required experienced laboratory professionals. Additionally, IFA detects the surface protein of DENV. At the time cell culture was performed, inoculation with low viral load present in dog serum samples, the concentration of DENV replicating inside the cells might be below the detection limit of IFA. RT-PCR is high sensitivity in detecting RNA replication in low viral load in the sample [46,52]. However, potential contamination during all steps of RT-PCR should be avoided.

The detection limit of nested RT-PCR, developed by Lanciotti et al., was 100 complete virus particles (36). Although all collected supernatants showed positive results by using nested RT-PCR, none of them showed positive result by in-house real-time PCR with LoD of 10^3^ copies/mL. Therefore, these samples may have the virus load at least 100 copies/mL but fewer than 1000 copies/mL. The reduction of virus particles contained in supernatant samples may have been the result of RNA degradation in the thawed samples. Hence, the storage history of samples should be of concern.

In the present study, DENV was successfully isolated from serum samples of domestic dogs living in a dengue endemic area. Considering the potential hosts of DENV, humans were the most likely ones [42]. However, domestic dogs could be candidate hosts, since they share habitats with humans, therefore, increasing opportunities for *Aedes* mosquito vectors to select them as hosts for feeding. Studies on the feeding pattern of *Aedes* spp. indicated that dogs were possible hosts. In rural settings of Singapore, *Ae*. *albopictus* were found to feed on both humans and dogs [34]. Likewise, in urban and suburban of northeastern USA, feeding on both humans and dogs was also reported [33]. Ponlawat et al. indicated that double-host blood meals including humans and dogs were found in both *Ae*. *aegypti* and *Ae*. *albopictus* collected from many regions of Thailand during 2003–2004 [25]. In addition, the study in rural Puerto Rico found 18–21% of *Ae*. *aegypti* fed on dogs. Our results showed that four out of seven PCR-positive dogs lived inside houses together with their owners. However, all of them had territories which did not extend far from the houses of their owners. A study on JEV in dogs in Bangkok, Thailand indicated that 36 out of 70 (51%) sampled dogs were tested sero-positive for JEV. In addition, 29 of 44 (66%) dogs which were kept outside houses were tested sero-positive for JEV; in contrast, all dogs which were kept inside houses were sero-negative [29]. These results were likely due to the fact that the main vector of JEV is *Culex* mosquitoes, which have been found mostly outside houses, while *Aedes* mosquitoes are found inside or around houses. However, a study on species and blood meal analysis of mosquitoes collected from Koh Chang, a tourist island of Thailand, indicated that the major blood meal of *Culex quinquefasiatus* was of dog origin (36%). Furthermore, they reported that *Ae*. *aegypti* also fed on domestic dogs [53].

We concluded that domestic dogs could be infected with DENV strains circulating in the endemic area. Likewise, studies on domestic dogs in relation to flavivirus surveillance, such as WNV, have stated that dogs can be infected by WNV and that they could be useful sentinels for monitoring areas for evidence of WNV [25–27,54] and JEV [28], which corresponds with a study that found antibodies against JE and dengue in serum collected from domestic dogs in Thailand [29]. The mild or subclinical symptoms that always show when these animals are infected with flavivirus diseases [21–30] could benefit the pathogens by perpetuating the virus infection in potential hosts and their vectors. Although the percentage of DENV-positive samples that we found is quite low, i.e., 0.95% in domestic dogs living in dengue urban area and 0.65% in rubber plantation area, this is the first evidence of dengue infection in domestic dogs. Further investigations on DENV and other mosquito-borne pathogens in domestic dogs should be carried out. Limitations of our study were based on the fact that this is the first study of DENV surveillance in dog populations in Thailand. High numbers of domestic dogs were required to test for the presence of DENV genome in their blood circulation. Qualitative PCR targeted on identification of highly conserved DENV sequences was used according to its high sensitivity and specificity of the method. However, the chance of contamination was strictly avoided. The RT-PCR used in this study produced small amplicons (119 bp for DENV serotype 2 and 290 bp for serotype 3 [36], making it impossible to discern relationships with other DENV strains circulating in the region. The study on the whole genome analysis of DENV isolated from domestic dogs should be performed in the future.

Although we have concluded that domestic dogs can be infected with DENV, the role of domestic dogs needs to be further investigated to identify whether they are potential reservoirs or incidental hosts. Surveillance of DENV in an endemic area should focus not only on human hosts and mosquito vectors, but also in domestic dog populations. This study provided an important clue for future effective dengue surveillance, prevention and control in dengue endemic areas in Thailand and elsewhere.

## Conclusion

Our study demonstrated the first evidence of dengue infections in domestic dogs living in an urban city and rubber plantation areas in Thailand. The overall infection frequency was quite low, 0.95% in the urban city vs 0.65% in the rubber plantation area. Further molecular analysis showed that two serotypes of DENVs, i.e., DENV serotype 2 and DENV serotype 3, were circulating in domestic dogs. Further research should focus on the role of domestic dogs in dengue transmission.

## References

[1] Gubler DJ. Dengue and dengue hemorrhagic fever. Clin Microbiol Rev. 1988;11: 258–61. 10.1128/cmr.11.3.480 PMC88892 9665979

[2] Sheperd DS, Undurraga EA, Halasa YA. Economic and disease burden of dengue in Southeast Asia. PLoS Negl Trop Dis. 2013;7: e2055 doi: 10.1371/journal.pntd.0002055 23437406 10.1371/journal.pntd.0002055 PMC3578748

[3] Bhatt S, Gething PW, Brady OJ, Messina JP, Farlow AW, Moyes CL, et al The global distribution and burden of dengue. Nature. 2013;496: 504–7. doi: 10.1038/nature12060 23563266 10.1038/nature12060 PMC3651993

[4] Aguiar M, Stollenwerk N, Halstead SB. The impact of the newly licensed dengue vaccine in endemic countries. PLoS Negl Trop Dis. 2016 12 21; 10(12): e0005179 doi: 10.1371/journal.pntd.0005179 28002420 10.1371/journal.pntd.0005179 PMC5176165

[5] Capeding MR, Tran NH, Hadinegoro SR, Ismail HI, Chotpitayasunondh T, Chua MN, et al Clinical efficacy and safety of a novel tetravalent dengue vaccine in healthy children in Asia: a phase 3, randomized observer-masked, placebo-controlled trial. Lancet. 2014;384: 1358–65. doi: 10.1016/S0140-6736(14)61060-6 25018116 10.1016/S0140-6736(14)61060-6

[6] Liu Y, Liu J, Cheng G. Vaccines and immunization strategies for dengue prevention. Emerg Microbes Infect. 2016;5: e77 doi: 10.1038/emi.2016.74 27436365 10.1038/emi.2016.74 PMC5141265

[7] Watanaveeradej V, Gibbons RV, Simasathien S, Nisalak A, Jarman RG, Kerdpanich A, et al Safety and immunogenicity of a rederived, lived-attenuated dengue virus vaccine in healthy adults living in Thailand: a randomized trial. Am J Trop Med Hyg. 2014;91: 119–28. doi: 10.4269/ajtmh.13-0452 24865677 10.4269/ajtmh.13-0452 PMC4080550

[8] Sirivichayakul C, Barranco-Santana EA, Esquilin-Rivera I, Oh HM, Raanan M, Sariol CA, et al Safety and immunogenicity of a tetravalent dengue vaccine (TDV) in healthy children and adults in endemic regions: a randomized, placebo-controlled Phase 2 study. J Infect Dis. 2016;213: 1562–72. doi: 10.1093/infdis/jiv762 26704612 10.1093/infdis/jiv762

[9] Little E, Barrera R, Seto KC, Diuk-Wasser M. Co-occurrence patterns of dengue vector *Aedes aegypti* and *Aedes mediovitattus*, a dengue competent mosquito in Puerto Rico. EcoHealth. 2011;8: 365–75. doi: 10.1007/s10393-011-0708-8 21989642 10.1007/s10393-011-0708-8 PMC4646052

[10] Holmes EC, Twiddy SS. The origin, emergence and evolutionary genetics of dengue virus. Infect Genet Evol. 2003;3: 19–28. 12797969 10.1016/s1567-1348(03)00004-2

[11] Weaver SC, Vasilakis N. Molecular evolution of dengue viruses: contributions of phylogenetics to understanding the history and epidemiology of the preeminent arboviral disease. Infect Genet Evol. 2009;9: 523–40. doi: 10.1016/j.meegid.2009.02.003 19460319 10.1016/j.meegid.2009.02.003 PMC3609037

[12] Rudnick A. Studies of the ecology of dengue in Malaysia: a preliminary report. J Med Entomol. 1965;2: 203–08. 5827577 10.1093/jmedent/2.2.203

[13] Kurane I. Dengue hemorrhagic fever with special emphasis on immuno-pathogenesis. Comp Immun Microbiol Infect Dis. 2007;30: 329–40. 10.1016/j.cimid.2007.05.010 17645944

[14] Althouse BM, Durbin AP, Hanley KA, Halstead SB, Weaver SC, Cummings D. Viral kinetic of primary dengue virus infection in non-human primates: a systematic review and individual pooled analysis. Virol. 2014;452–453: 237–46. 10.1016/j.virol.2014.01.015 PMC4578724 24606701

[15] Zhang H, Yang X, Li G. Detection of dengue virus genome RNA in some kinds of animals caught from dengue fever endemic areas in Hainan Island with reverse transcription polymerase chain reaction. Zhonghua Shi Yan He Lin Chuang Bing Du Xue Za Zhi. 1998;3: 226–28. 12526321

[16] Aguilar-Setien A, Romeo-Almaraz M, Sánchez-Hernández C, Figueroa R, Juárez-Palma LP, Garcia-Flores MM, et al Dengue in Mexican bats. Epidemiol Infect. 2008;136: 1678–83. doi: 10.1017/S0950268808000460 18325131 10.1017/S0950268808000460 PMC2870782

[17] Sotomayor-Bonilla J, Chaves A, Rico-Chávez O, Rostal MK, Ojeda-Flores R, Salas-Rojas M, et al Short report: Dengue in bats from Southeastern Mexico. Am J Trop Med Hyg. 2014;91: 129–31. doi: 10.4269/ajtmh.13-0524 24752688 10.4269/ajtmh.13-0524 PMC4080551

[18] Platt K, Mangiafico J, Rocha O, Zaldivar M, Mora J, Trueba G, et al Detection of dengue virus neutralizing antibodies in bats from Costa Rica and Ecuador. J Med Entomol. 2000;37: 965–67. 11126559 10.1603/0022-2585-37.6.965

[19] Machain-Williams C, López-Uribe M, Talavera-Aguilar L, Carrillo-Navarrete J, Vera-Escalante L, Puerto-Manzano F, et al Serological evidence of flavivirus infection in bats in the Yucatan Peninsula of Mexico. J Wildl Dis. 2013;49: 684–89. doi: 10.7589/2012-12-318 23778622 10.7589/2012-12-318 PMC3864018

[20] De Thoisy B, Dussart P, Kazanji M. Wild terrestrial rainforest mammals as potential reservoirs for flaviviruses (yellow fever, dengue 2 and St. Louis encephalitis viruses) in French Guiana. Trans R Soc Trop Med Hyg. 2004;7: 409–12. 10.1016/j.trstmh.2003.12.003 15138077

[21] De Thoisy B, Lacoste V, Germain A, Munoz-Jordan J, Colon C, Mauffrey J, et al Dengue infection in neotropical forest mammals. Vector Borne Zoonotic Dis. 2009;2: 157–70. 10.1089/vbz.2007.0280 18945183

[22] Lavergne A, Lacoste V, Germain A, Matheus S, Dussart P, Deparis X, de Thoisy B. Dengue virus infection in neotropical forest mammals: incidental hosts or potential reservoirs? Med Trop (Mars). 2009;4: 345–50. 19725384

[23] Wolfe ND, Kilbourn AM, Karesh WB, Rahman HA, Bosi EJ, Cropp BC, et al Sylvatic transmission of arboviruses among Bornean orangutans. Am J Trop Med Hyg 2001;64: 310–16. 11463123 10.4269/ajtmh.2001.64.310

[24] Inoue S, Morita K, Matias RR, Tuplano JV, Resuello PR, Candelario JR, et al Distribution of three arboviruses antibodies among monkey (*Macaca fascicularis*) in the Philippines. J Med Primatol. 2003;32: 89–94. 12823631 10.1034/j.1600-0684.2003.00015.x

[25] Kile JC, Panella NA, Komar N, Chow CC, MacNeil A, Robbins B. Serological survey of cats and dogs during an epidemic of West Nile virus in humans. J Am Vet Med Assoc. 2005;226: 1349–53. 15844427 10.2460/javma.2005.226.1349

[26] Resnick MP, Grunenwald P, Blackmar D, Hailey C, Bueno R, Murray KO. Juvenile dogs as potential sentinels for West Nile virus surveillance. Zoonoses Public Health. 2008;55: 443–47. doi: 10.1111/j.1863-2378.2008.01116.x 18399945 10.1111/j.1863-2378.2008.01116.x

[27] Lan D, Ji W, Yu D, Chu J, Wang C, Yang Z, et al Serological evidence of West Nile virus in dogs and cats in China. Arch Virol. 2011;156: 893–95. doi: 10.1007/s00705-010-0913-8 21221671 10.1007/s00705-010-0913-8

[28] Shimoda H, Ohno Y, Mochizuki M, Iwata H, Okuda M, Maeda K. Dogs as sentinels for human infection with Japanese encephalitis virus. Emerg Infect Dis. 2010;16: 1137–39. doi: 10.3201/eid1607.091757 20587189 10.3201/eid1607.091757 PMC3321903

[29] Shimoda H, Inthong N, Noguchi K, Terada Y, Nagao Y, Shimojima M, et al Development and application of an indirect enzyme-linked immunosorbent assay for serological survey of Japanese encephalitis virus in dogs. J Virol Methods. 2013;187: 85–89. doi: 10.1016/j.jviromet.2012.09.022 23046992 10.1016/j.jviromet.2012.09.022

[30] Calvignac-Spencer S, Leendertz SAJ, Gillespie TR, Leendertz FH. Wild great apes as sentinels and sources of infectious disease. Clin Microbiol Infect. 2012;18: 521–27. doi: 10.1111/j.1469-0691.2012.03816.x 22448813 10.1111/j.1469-0691.2012.03816.x

[31] Ponlawat A, Harrington LC. Blood feeding patterns of *Aedes aegypti* and *Aedes albopictus* in Thailand. J Med Entomol. 2005;42: 844–49. 16363170 10.1093/jmedent/42.5.844

[32] Barrera R, Bingham AM, Hassan HK, Amador M, Mackay AJ, Unnasch TR. Vertebrate hosts of *Aedes aegypti* and *Aedes mediovittatus* (Diptera: Culicidae) in Rural Puerto Rico. J Med Entomol. 2012;49: 917–21. 22897052 10.1603/me12046 PMC4627690

[33] Faraji A, Egizi A, Fonseca DM, Unlu I, Crepeau T, Healy SP, et al Comparative host feeding patterns of the Asian tiger mosquito, *Aedes albopictus*, in urban and suburban Northeastern USA and implications for disease transmission. PLoS Negl Trop Dis. 2014;8: e3037 doi: 10.1371/journal.pntd.0003037 25101969 10.1371/journal.pntd.0003037 PMC4125227

[34] Gaunt MW, Sall AA, de Lamballerie X, Falconar AK, Dzhivanian TI, Gould EA. Phylogenetic relationships of flaviviruses correlate with their epidemiology, disease association and biogeograpgy. J Gen Virol. 2001;82: 1867–76. doi: 10.1099/0022-1317-82-8-1867 11457992 10.1099/0022-1317-82-8-1867

[35] Kek R, Hapuarachchi HC, Chung C, Humaidi MB, Razak MA, Chiang S, et al Feeding host range of *Aedes albopictus* (Diptera: Culicidae) demonstrates its opportunistic host-seeking behavior in rural Singapore. J Med Entomol. 2014;51: 881–84. 10.1603/me13213 25118424

[36] Lanciotti RS, Calisher CH, Gubler DJ, Chang G-J, Vorndam V. Rapid detection and typing of dengue viruses from clinical samples by using reverse transcriptase-polymerase chain reaction. J Clin Microbiol. 1992;87: 873–83. 10.1128/jcm.30.3.545-551.1992 PMC265106 1372617

[37] Thompson JD, Higgins DG, Gibson TJ. CLUSTAL W: improving the sensitivity of progressive multiple sequence alignment through sequence weighting, position-specific gap penalties and weight matrix choice. Nucleic Acids Res. 1994;22: 4673–80. 7984417 10.1093/nar/22.22.4673 PMC308517

[38] Tamura K. Stecher G, Peterson D, Fillipski A, Kumar S. Mega6: Molecular Evolutionary Genetics Analysis version 6.0. Mol Biol Evol. 2013;30: 2725–9. doi: 10.1093/molbev/mst197 10.1093/molbev/mst197 PMC3840312 24132122

[39] Dengue haemorrhagic fever: diagnosis, treatment, prevention and control, 1997 2^nd^ edition Geneva: World Health Organization. 23762963

[40] De Paula SO, Pires Neto RJ, Tocantins Correa JA, Asummpcao SR, Costa MLS, Lopes Fonseca BA. The use of reverse transcription-polymerase chain reaction (RT-PCR) for the rapid detection and identification of dengue virus in an endemic region: a validation study. Trans R Soc Trop Med Hyg. 2002;96: 266–69. 12174774 10.1016/s0035-9203(02)90094-5

[41] Roehrig J. Guidelines for plaque reduction neutralizing testing of human antibodies to dengue viruses. World Health Organization. 2007. 10.1089/vim.2008.0007 18476771

[42] De Paula SO, Fonseca BAL. Dengue: a review of the laboratory tests a clinician must know to achieve a correct diagnosis. BJID. 2004;8: 390–98. 10.1590/s1413-86702004000600002 15880229

[43] Lee E, Nestorowicz A, Marshall ID, Weir RC, Dalgarno L. Direct sequence analysis of amplified dengue virus genomic RNA from cultured cells, mosquitoes and mouse brain. J Virol Methods. 1992;37: 275–88. 1634599 10.1016/0166-0934(92)90029-d

[44] Robertson ID, Irwin PJ, Lymbery AJ, Thomson RCA. The role of companion animals in the emergence of parasitic zoonoses. Int J Parasitol. 2000;30: 1369–77. 11113262 10.1016/s0020-7519(00)00134-x

[45] Conceicao TM, Da Poian AT, Sorgine M. A real–time PCR procedure for detection of dengue virus serotypes 1, 2, and 3 and their quantitation in clinical and laboratory samples. J Virol Methods. 2010;163: 1–9. doi: 10.1016/j.jviromet.2009.10.001 19822173 10.1016/j.jviromet.2009.10.001

[46] Jarman RG, Nisalak A, Anderson KB, Klungthong C, Thaisomboonsul B, Kaneechit W, et al Factors influencing dengue virus isolation by C6/36 cell culture and mosquito inoculation of nested PCR-positive clinical samples. Am J Trop Med Hyg. 2011;84: 218–23. doi: 10.4269/ajtmh.2011.09-0798 21292887 10.4269/ajtmh.2011.09-0798 PMC3029170

[47] Samuel PP, Tyagi BK. Diagnostic methods for detection and isolation of dengue viruses from vector mosquitoes. Indian J Med Res. 2006;123: 615–28. 16873905

[48] Azhar E, Kao M, Niedrig M, Masri B, Godus A, Badierah R. Virological diagnosis of dengue fever in Jeddah, Saudi Arabia: comparison between RT-PCR and virus isolation in cell culture. J Infect Dis Immun. 2010;2: 24–29.

[49] Sa-ngasang A, Wibulwattanakij S, Chanama S, O-rapinpatipat A, A-nuegoonpipat A, Anantaprecha S, et al Evaluation of RT-PCR as a tool for diagnosis of secondary dengue virus infection. Jpn J Infect Dis. 2003;56: 205–09. 14695431

[50] Pinheiro V, Tadei WP, Barros PM, Vasconcelos P, Cruz AC. Detection of dengue virus serotype 3 by reverse transcription in *Aedes aegypti* (Diptera, Culicidae) captures in Manaus, Amazonas. Mem Ins oswaldo Cruz, Rio de Janeiro. 2005;100: 833–39. 10.1590/s0074-02762005000800003 16444413

[51] Barkham TM, Chung YK, Tang HF, Ooi EE. The performance of RT-PCR compared with a rapid serological assay for acute dengue fever in a diagnostic laboratory. Trans R Soc Trop Med Hyg. 2006;100: 142–48. doi: 10.1016/j.trstmh.2005.05.015 16212996 10.1016/j.trstmh.2005.05.015 PMC7107224

[52] De Paula SO, Lima DM, Clotteau M, Pires Neto RJ, Fonseca BAL. Improved detection of dengue-1 virus from IgM-positive serum samples using C6/36 cell cultures in association with RT-PCR. Intervirology. 2003;46: 227–31. 12931031 10.1159/000072432

[53] Khaklang S, Kittayapong P. Species composition and blood meal analysis of mosquitoes collected from a tourist island, Koh Chang, Thailand. J Vector Ecol. 2014;39: 448–52. doi: 10.1111/jvec.12122 25424275 10.1111/jvec.12122

[54] Shen SH, Stauft CB, Gorbatsevych O, Song Y, Ward CB, Yurovsky A, et al Large-scale recoding of an arbovirus genome to rebalance its insect versus mammalian preference. PNAS. 2015;112: 4749–54. doi: 10.1073/pnas.1502864112 25825721 10.1073/pnas.1502864112 PMC4403163

